# Cytosolic Double-Stranded DNA as a Damage-Associated Molecular Pattern Induces the Inflammatory Response in Rat Pancreatic Stellate Cells: A Plausible Mechanism for Tissue Injury-Associated Pancreatitis

**DOI:** 10.1155/2012/504128

**Published:** 2012-04-09

**Authors:** Taichi Nakamura, Tetsuhide Ito, Hisato Igarashi, Masahiko Uchida, Masayuki Hijioka, Takamasa Oono, Nao Fujimori, Yusuke Niina, Koichi Suzuki, Robert T. Jensen, Ryoichi Takayanagi

**Affiliations:** ^1^Department of Medicine and Bioregulatory Science, Graduate School of Medical Sciences, Kyushu University, 3-1-1 Maidashi, Higashi-ku, Fukuoka 812-8582, Japan; ^2^Leprosy Research Center, National Institute of Infectious Diseases, Tokyo 189-0002, Japan; ^3^Cell Biology Section, NIDDK, National Institutes of Health, Bethesda, MD 20892, USA

## Abstract

Pancreatitis is an inflammatory disease of unknown causes. There are many triggers causing pancreatitis, such as alcohol, common bile duct stone, virus and congenital or acquired stenosis of main pancreatic duct, which often involve tissue injuries. Pancreatitis often occurs in sterile condition, where the dead/dying pancreatic parenchymal cells and the necrotic tissues derived from self-digested-pancreas were observed. However, the causal relationship between tissue injury and pancreatitis and how tissue injury could induce the inflammation of the pancreas were not elucidated fully until now. This study demonstrates that cytosolic double-stranded DNA increases the expression of several inflammatory genes (cytokines, chemokines, type I interferon, and major histocompatibility complex) in rat pancreatic stellate cells. Furthermore, these increase accompanied the multiple signal molecules genes, such as interferon regulatory factors, nuclear factor-kappa B, low-molecular-weight protein 2, and transporter associated with antigen processing 1. We suggest that this phenomenon is a plausible mechanism that might explain how cell damage of the pancreas or tissue injury triggers acute, chronic, and autoimmune pancreatitis; it is potentially relevant to host immune responses induced during alcohol consumption or other causes.

## 1. Introduction

In 1998, star-shaped cells in the pancreas called pancreatic stellate cells (PSCs) were identified and characterized [1, 2]. In a normal pancreas, PSCs are quiescent and can be identified by the presence of vitamin A-containing lipid droplets in the cytoplasm. In response to pancreatic injury or inflammation, they are transformed from their quiescent phenotype into myofibroblast-like cells, which actively proliferate, express *α*-smooth muscle actin (*α*-SMA), and produce extracellular matrix components such as type I collagen [3–5]. Although the transition from quiescent to activated PSCs is triggered by various types of molecules, recent evidence suggests that components of dead/dying host cells may also trigger this transition [6].

This study aimed to determine whether host double-stranded DNA (dsDNA) contributes to the functions of PSCs, particularly in inflammation. Although DNA was historically believed to be immunologically inert, it is now appreciated that DNA can be recognized by the immune system [7, 8]. For example, unmethylated CpG motifs, which are expressed at high frequency in bacterial DNA, cause lymphocytes, dendritic cells, and pancreatic stellate cells to proliferate and secrete immunoglobulin and/or cytokines [9–11], and dsDNA upregulates surface expression of major histocompatibility complex (MHC) molecules in thyroid cells [12]. Although DNA is normally sequestered in the nucleus, it can be released into the systemic circulation when cells undergo necrosis/apoptosis. Exposure to DNA has been implicated in the development of autoimmune and inflammatory diseases and has been observed in DNase-deficient mice [13]. These findings led us to hypothesize that dsDNA released by injured host cells may act as a “danger signal,” which affects PSCs.

Here, we report that cytosolic dsDNA induces the expression of various inflammatory genes, which play a role in the tissue damage that mediates the inflammatory activity of host dsDNA.

## 2. Materials and Methods

### 2.1. Materials

Poly (dA : dT), Poly (dI : dC), mouse antirat alpha-smooth muscle actin antibody, and lipofectamine 2000 were obtained from Sigma-Aldrich (St. Louis, MO, USA). Antimouse IgG Alexa 555-conjugated antibody was obtained from Invitrogen (Carlsbad, CA, USA).

### 2.2. Isolation of PSCs and Cell Culture

PSCs were isolated from male Wistar rats by density-gradient centrifugation method. Cells were maintained in complete DMEM/F-12: this is a mixture of DMEM (Dulbecco's modified Eagle medium) and Ham's F-12 nutrient mixture supplemented with 10% fetal bovine serum, 50 units/mL of penicillin, and 50 mg/mL of streptomycin (all from Invitrogen, Carlsbad, CA, USA). All experiments were performed with cells between passages three and four. Unless specifically described, we incubated PSCs in serum-free medium for 24 h before the addition of experimental reagents. All animal procedures were performed in accordance with the guidelines of the Committee on Animal Care of the Kyushu University.

### 2.3. Transfection

Unless otherwise noted, 10 *μ*g of DNA was mixed with 5 *μ*L of Lipofectamine2000 and 985 *μ*L of serum-free medium and then incubated for 15 min at room temperature. A duplicate mixture without DNA and/or lipofectamine2000 also was incubated for 15 min at room temperature. Cells were washed with serum-free medium, and the combined mixtures were added for DNA transfection (Tfx).

### 2.4. Expression of Cytosolic DNA Receptors and the Effects of dsDNA on the Functions of PSCs: Real-Time Reverse-Transcription Polymerase Chain Reaction

Total RNA was extracted from PSCs using an RNeasy mini kit (Qiagen, Valencia, CA, USA). For RT-PCR, 20–100 ng of total RNA was reverse transcribed into first-strand complementary DNA (cDNA) using a PrimeScript RT reagent kit (Takara Bio, Inc, Otsu, Shiga, Japan) according to the manufacturer's instructions. RT-PCR was performed using a LightCycler Real-Time PCR system (Roche, Switzerland) according to the manufacturer's instructions. The reaction mixture (20 *μ*L) contained SYBR Premix Ex Taq II (TLi RNAseH Plus; Takara Bio, Inc, Otsu, Shiga, Japan), 4 mM MgCl_2_, 0.5 mM of the upstream and downstream PCR primers (Table 1), and 2 *μ*L of first-strand cDNA template. To control for variations in the reactions, all PCR data were normalized against GAPDH expression.

### 2.5. Quantification of Soluble Monocyte Chemoattractant Protein-1(MCP-1): MCP-1 ELISA

After 24 h of incubation, the levels of MCP-1 in the culture supernatants were measured by ELISA (Rat MCP-1 ELISA from Thermo Scientific, Rockford, IL, USA) according to the manufacturers' instructions.

### 2.6. Cell Viability Assay: MTS Assay

Cell viability was assessed by the MTS assay (CellTiter 96 Aqueous One Solution Cell Proliferation Assay, Madison, WI, USA). After treatment with dsDNA for 24 h, MTS solution was added to the cells and the incubation continued at 37°C for 1 h. After the incubation period, cell viability was quantified by the differences in absorbance at wavelengths of 570 and 690 nm.

### 2.7. Cell Cytotoxicity Assay: LDH Assay

Cell cytotoxicity was assessed by the LDH assay (CytoTox 96 Non-Radioactive Cytotoxicity Assay, Madison, WI, USA). After treatment with dsDNA for 24 h, the cell supernatants were transferred to another microplate, and then LDH substrate was added to the supernatants and the incubation continued at 37°C for 30 min. After the incubation period, stop solution was added and cell cytotoxicity was quantified by the differences in absorbance at wavelengths of 570 and 690 nm.

### 2.8. Expression of Alpha-Smooth Muscle Actin and M30 Cytokeratin: Immunofluorescent Confocal Microscopy

Cell activation and cell apoptosis was assessed by immunofluorescent cytochemistry. Mouse antirat alpha-smooth muscle actin antibody were used to evaluate cell activation, and FITC-labelled M30 antibody was used to evaluate apoptosis. After incubation, cells were washed with phosphate-buffered saline, fixed in 4% paraformaldehyde, and analyzed for fluorescence under a confocal laser scanning microscope (Nikon A1/C1, Tokyo, Japan).

### 2.9. Statistical Analysis

Results are expressed as the means (SEM) of 3-4 separate cell preparations per experimental protocol. Student's *t-*test was used for the statistical analyses. *P* values of <0.05 were considered statistically significant.

## 3. Results

### 3.1. Rat PSCs Expressed Cytosolic DNA Receptors

**Figure 1:**
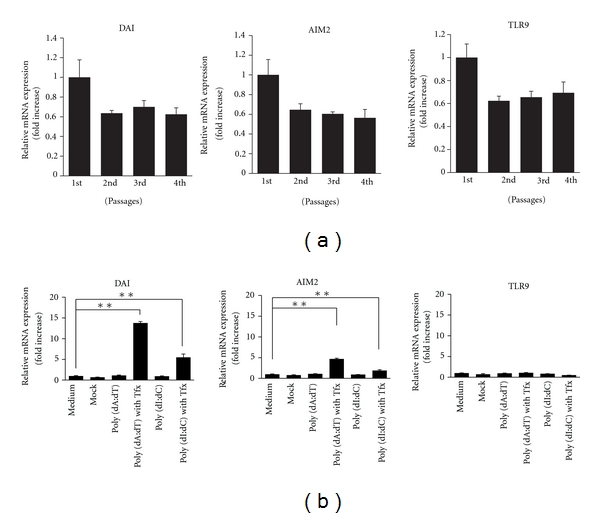
(a) Pancreatic stellate cells (PSCs) expressed double-stranded DNA (dsDNA) receptors. Total RNA was prepared from freshly isolated (3 days after isolation) culture-activated PSCs (passages 2 and 4). Expression of the dsDNA receptors was assessed by real-time PCR. All PSCs constitutively expressed DNA-dependent activator of IFN-regulatory factors (DAI), absent in melanoma 2 (AIM2), and toll-like receptor 9 (TLR9). (b) Extracellular DNA stimulation had no effect on DNA receptors, such as DAI and AIM2. In contrast, intracellular dsDNA increased the expression of all dsDNA receptors except TLR9. PSCs: pancreatic stellate cells, TFx: + transfection reagent lipofectamine. *P < 0.05, **P < 0.01.

There have been no previous reports on the expression of foreign DNA receptors in PSCs other than toll-like receptor 9 (TLR9) [2]. Therefore, we first measured the mRNA expression of DNA-dependent activator of IFN-regulatory factors (DAI) and absent in melanoma 2 (AIM2), which recognize cytosolic dsDNA using real-time PCR. PSCs expressed both the DAI and AIM2 receptors regardless of the passage and could recognize cytosolic DNA (Figure 1(a)). Next, synthesized dsDNA was introduced into the cytoplasm by lipofection to determine whether the number of receptors increased in response, that is, whether inflammation was initiated. The synthesized dsDNA used in this study had a structure similar to that of host dsDNA, and has been widely used to imitate host dsDNA that is derived from cell and tissue injury. Poly (dA : dT) has been reported to induce type I interferon (IFN) cytokines, and chemokines, and triggers the inflammatory response. However, it is also known that dsDNA is transformed into double-stranded RNA (dsRNA) by RNA polymerase III and is detected by the RIG-I receptor, which recognizes dsRNA. Therefore, this is not true DNA stimulation [14]. In contrast, poly (dI : dC) lacks the 3′-ppp structure that is sensed by RIG-I and is sensed only by receptors that recognize dsDNA. Although poly (dA : dT) and poly (dI : dC) are not influenced by extracellular DNA stimulation, introduction of intracellular dsDNA by lipofection has been shown to significantly increase the number of the receptor expression and induce inflammation (Figure 1(b)).

### 3.2. dsDNA Increased Cytokine and Chemokine Expression

**Figure 2:**
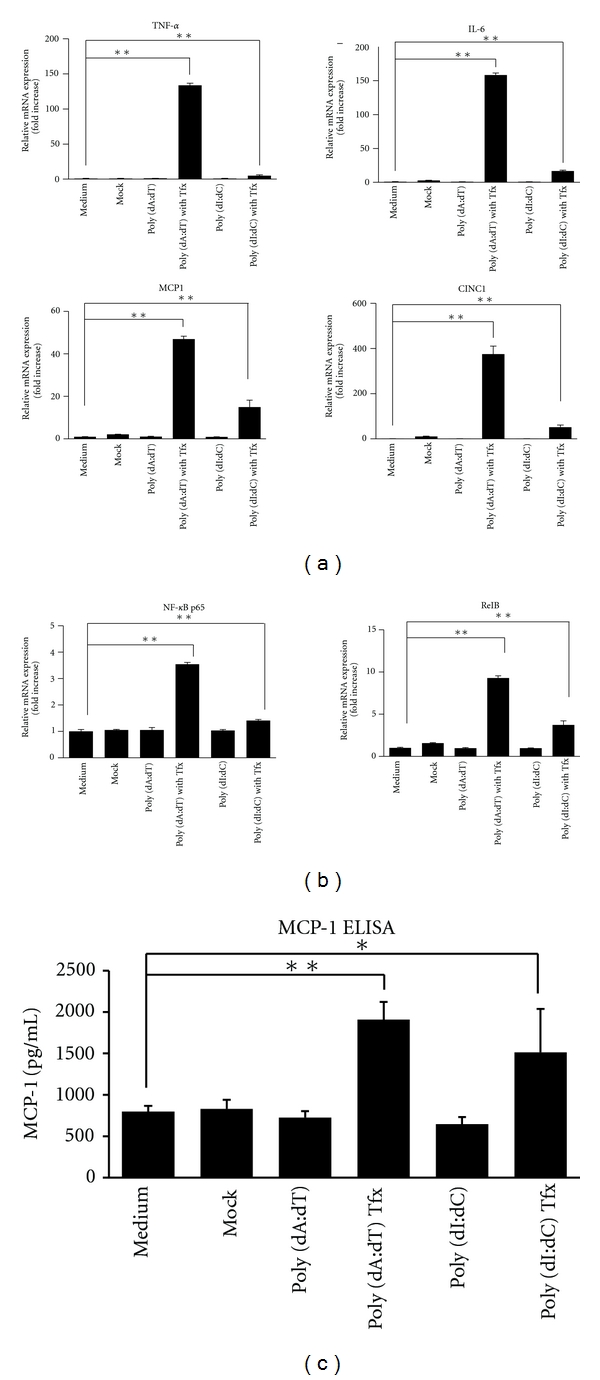
Transcription of cytokine and chemokine mRNA was induced by synthetic double-stranded DNA (dsDNA). (a) Extracellular DNA stimulation had no effect on inflammatory cytokines and chemokines, such as tumor necrosis factor-alpha (TNF-*α*), interleukin-6 (IL-6), monocyte chemoattractant protein-1 (MCP-1), and cytokine-induced neutrophil chemoattractant 1 (CINC-1). In contrast, intracellular dsDNA (at 10 *μ*g/mL) had stimulatory effects on their expression, including the expression of their transcriptional factors nuclear factor-kappa B (NF-*κ*B) and reticuloendotheliosis viral oncogene homolog B (RelB) (b). Release of MCP-1 was also confirmed by ELISA (c). PSCs: pancreatic stellate cells, TFx: + transfection reagent lipofectamine. *P < 0.05, **P < 0.01.

Next, we determined whether the expression of inflammatory cytokine and chemokine genes was induced using RT-PCR. The results indicated that although extracellular DNA stimulation did not induce expression of proinflammatory cytokines such as tumor necrosis factor-alpha (TNF-*α*) and interleukin-6 (IL-6) and chemokines such as MCP-1 and cytokine-induced neutrophil chemoattractant 1 (CINC-1), intracellular dsDNA did stimulate their expression at 6 h (Figure 2(a)). Gene expression is regulated by transcription factors such as nuclear factor-kappa B (NF-*κ*B). This study showed that the expression of such genes was increased by intracellular dsDNA stimulation (Figure 2(b)), which suggested that release of excess host dsDNA due to viral infection and tissue injury might trigger inflammation.

### 3.3. dsDNA Induced MHC Expression

**Figure 3:**
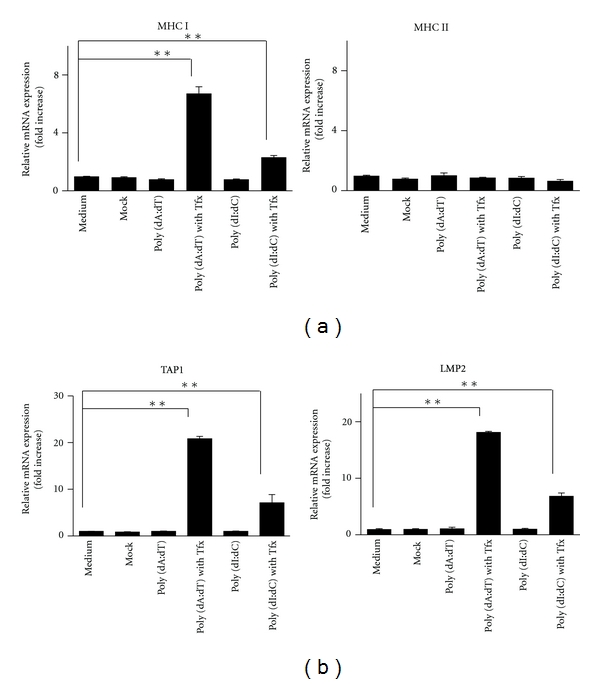
Major histocompatibility complex (MHC) mRNA transcription is induced by synthetic double-stranded DNA (dsDNA). (a) Extracellular DNA (at 10 *μ*g/mL) stimulation has no effect on MHC class I and class II. In contrast, intracellular dsDNA (at 10 *μ*g/mL) increased expression of their transcriptional factors transporter associated with antigen processing 1 (TAP1) and low-molecular-weight protein 2 (LMP2) (b). TFx: + transfection reagent lipofectamine. *P < 0.05, **P < 0.01.

We also determined the presence or absence of expression of gene-controlled antigen presentation, which activates T-cell-mediated cellular immunity. The results revealed that intracellular dsDNA stimulation increased MHC class I gene expression and was involved in not only the inflammation but also the activation of lymphocytes and others (Figure 3(a)). MHC class II expression was also examined because PSCs reportedly have phagocytic activity [15]; however, the expression was not increased. Transporter associated with antigen processing 1 (TAP1) and low-molecular-weight protein 2 (LMP2) play an important role in the induced expression of MHC class I [12]. Our study showed that TAP1 and LMP2 expression also increased (Figure 3(b)), which suggested that the presence of excess host dsDNA due to tissue injury might induce the abnormal MHC expression observed in patients with both chronic and autoimmune pancreatitis.

### 3.4. dsDNA Induced Type I IFN Induction

**Figure 4:**
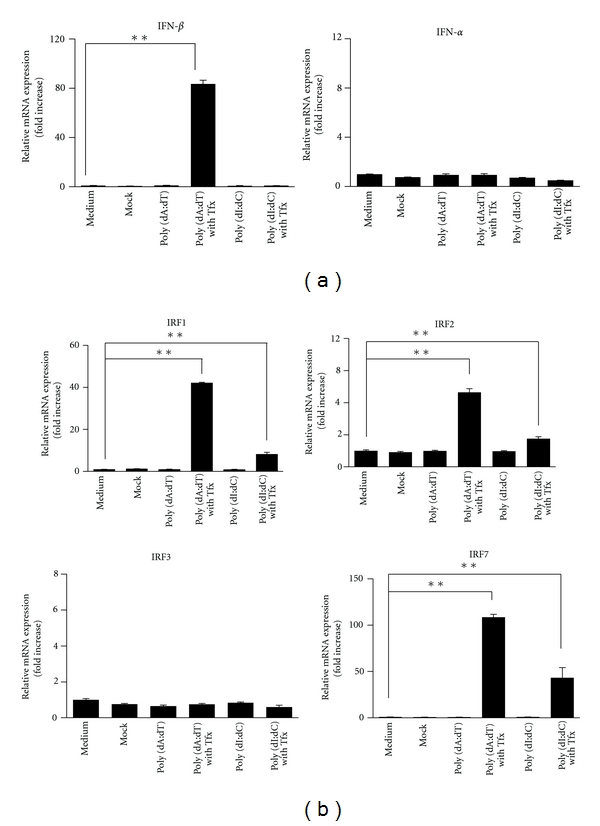
Transcription of type I interferon mRNA is induced by synthetic double-stranded DNA (dsDNA). (a) Extracellular DNA stimulation had no effect on the induction of type I interferons (IFNs), such as IFN-*α* and IFN-*β*. In contrast, intracellular dsDNA (at 10 *μ*g/mL) has stimulatory effects on their expression, including the expression of their transcriptional factors interferon regulatory factor 1 (IRF1), IRF2, and IRF7 (b). TFx: + transfection reagent lipofectamine. *P < 0.05, **P < 0.01.

Like MHC, type I IFN is involved in the activation of cell-mediated immunity; either IFN-*α* or IFN-*β* is predominantly induced depending on the cell type. In case of PSCs, IFN-*β* induction, which has also been reported in fibroblasts, has been observed (Figure 4(a)). Various interferon regulatory factors (IRFs) are involved in the expression of the above-mentioned genes [12]. In this study, the expression of IRF 1, 2, and 7 increased, while IRF3 expression was not induced (Figure 4(b)).

### 3.5. dsDNA Impaired Cell-Specific Functions

**Figure 5:**
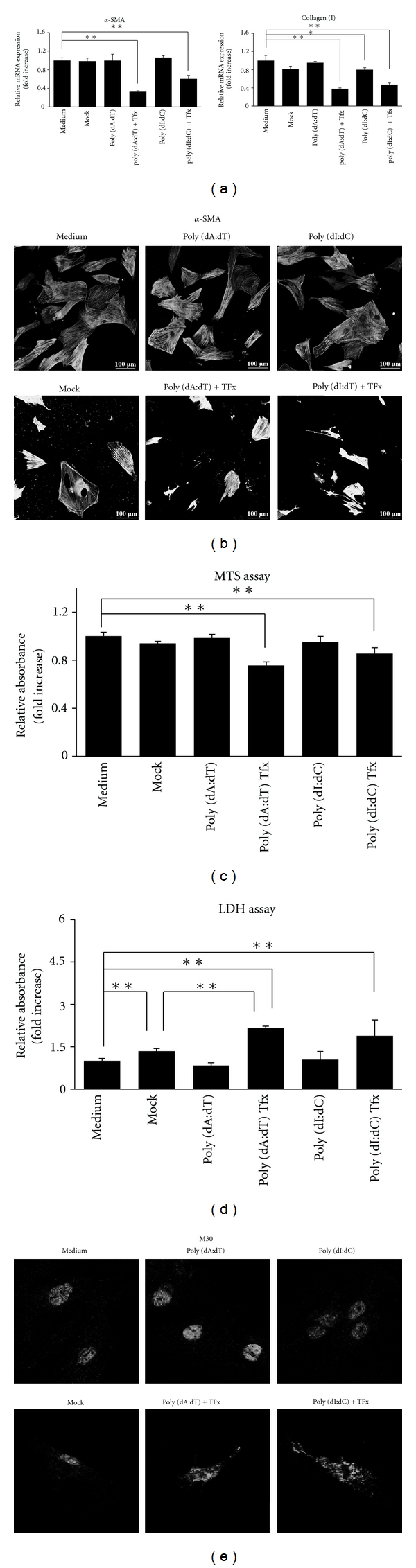
Double-stranded DNA (dsDNA) impaired cell-specific function. Cell-specific functions were impaired by intracellular dsDNA (at 1–10 *μ*g/mL) and extracellular poly (dI : dC) (at 10 *μ*g/mL) (a), (b). Intracellular dsDNA (at 10 *μ*g/mL) attenuated cell proliferation of PSCs (c)–(e). TFx: + transfection reagent lipofectamine. *P < 0.05, **P < 0.01.

It has been reported that engulfment of necrotic acinar cells attenuated the activation and collagen synthesis of PSC [6]. We examined whether this phenomenon was reproducible when PSCs were stimulated with synthetic dsDNA. Intracellular dsDNA attenuated activation, type I collagen gene induction and proliferation (Figures 5(a) and 5(c)). Furthermore, extracellular poly (dI : dC) attenuated type I collagen gene induction, indicating the possible function of extracellular dsDNA. We confirmed the decrease of *α*-SMA at protein level by immunofluorescent cytochemistry (Figure 5(b)). LDH assay and M30 staining revealed the concomitance of cell death, including necrosis and apoptosis (Figures 5(d) and 5(e)).

## 4. Discussion

At the time of their discovery, PSCs were identified as fibroblasts that maintained the homeostasis of the extracellular matrix [1, 2]. However, PSCs have recently been recognized as a multifunctional cell type [5, 16–18] that differ slightly from the well-differentiated cells of the pancreas, such as acinar cells, duct cells, and endocrine cells. The innate immune response of PSCs is particularly important in the induction of pancreatic inflammation. Furthermore, phagocytosis of various extracellular bacteria and dead cells by PSCs leads to activation of cellular immunity through antigen presentation, and there have been some reports on phagocytosis and endocytosis by PSCs [15, 17]. Stimulation of the innate immunity is generally divided into infectious and noninfectious stimulation [19], and the pathways that sense the stimulation include the phagolysosomal pathway, the endosomal-lysosomal pathway, and other autophagy pathways that function through phagocytosis. Some types of stimulation are recognized by diverse nucleic acid receptors that induce inflammatory responses through activation of common downstream transcriptional factors such as NF-*κ*B and IRF [20, 21]. In contrast, there are pathological conditions that induce inflammatory responses in an uninfected environment, which is similar to the infectious environment. The mechanism of inflammation was previously unknown. However, it is currently widely understood that inflammatory diseases develop because the innate immunity is activated by endogenous molecules released as a result of tissue injury, which are referred to as damage-associated molecular patterns (DAMPs) [22, 23].

The release of DAMPs is induced by various tissue injuries, for example, ischemia and reperfusion injury [24], trauma [25], and other harmful stimulations (alcoholic pancreatitis, drug-induced pancreatitis, and others) that induce critical apoptosis and necrosis. As a result, tissue injury induces the release of intracellular molecules (nucleic acids, HSP, UA, HMGB1, and others) and the degradation of extracellular matrix (hyaluronic acid), which results in induction of inflammation and repair of the injured sites [26]. However, uncontrolled tissue injury, for example, autodigestion caused by massive necrosis of pancreatic acinar cells, produces many necrotic cells. DAMPs, such as genomic DNA fragments, activate innate immunity and acquired immunity and induce autoimmune inflammation while inhibiting cell-specific functions [12, 27, 28]. Dead cells are usually removed by resident phagocytes; however, the pancreas does not have interstitial macrophages (*Mϕ*) similar to hepatic Kupffer cells, and so the PSCs phagocytose the dead cells. Therefore, PSCs are considered to be the primary cells that induce inflammation. The intrahepatic *Mϕ* of DNase II knock-out mice lack the ability to degrade the nucleic acids of apoptotic phagocytosis and produce many type I IFNs, which leads to chronic inflammation [13]. DNase I knockout mice develop antinuclear antibody-positive SLE-like symptoms through this same mechanism [29], and the intracellular DNA receptors in DNase III knock-out mice are activated by the accumulation of extranuclear DNA, which results in the onset of lethal inflammatory myocarditis associated with massive IFN induction [30]. The above-mentioned findings demonstrate that excessive nucleic acid accumulation from dead cells induces breakdown of the intracellular DNA processing system. This leads to the production of cytosolic dsDNA, which normally does not exist and not only triggers inflammation but also leads to the development of autoimmune disease. Furthermore, these findings suggest that the processing mechanism of DNase as well as its stimulation by dsDNA should be studied.

Most previous studies on the innate immune response in PSCs have focused only on infectious stimulation, and there have been no reports on DAMPs. Although it is known that an increase in the number of receptors due to stimulation by PAMPs or DAMPs primes inflammation, the change in the number of receptors expressed, such as the TLRs of PSCs, has not been studied. There are at least 2 receptors that recognize intracellular dsDNA and trigger inflammation. In particular, the expression of DAI and AIM2, which are most responsible for the inflammation, increased (Figure 1(b)), which suggests that a minor tissue injury could spread to entire organ [31, 32]. However, the number of TLR9 receptors did not increase. Instead, receptors that are specifically sensitive to certain stimulation increased, which could differentiate between self and nonself. In addition to the 2 above-mentioned receptors, there are other nucleic acid receptors, and extracellular H2B is thought to play an important role in the onset of autoimmune thyroid disease [27, 33, 34]. Which receptor or receptors are responsible for the onset of pancreatitis should be clarified in the future. The induction of cytokines and chemokines has a significant effect on the onset and clinical cure of pancreatitis. There have been reports that stimulation by a component of gram-negative bacilli, such as LPS or flagellin, a component of gram-positive cocci, such as LTA, or stimulation similar to that in viral infection, such as Poly (I : C), has effects similar to stimulation by cytosolic dsDNA. Therefore, common transcriptional factors, such as NF-*κ*B, are thought to induce expression of inflammatory cytokine and chemokine genes [17, 35, 36]. We previously reported that dsDNA from bacteria such as *Escherichia* *coli* has no inflammatory effect [11]. This may be because the intracellular DNA receptors are not stimulated due to various factors, including the length and amount of bacterial DNA and the cellular uptake pathway for the bacterial genomic DNA. In this study, we demonstrated for the first time that expression of cellular immunity activation factors associated with antigen presentation, such as IFN-*β*, and MHC class I, were induced by cytosolic dsDNA stimulation. We consider that these data will be useful for the evaluation of aberrant MHC expression and lymphocyte activation in the pancreatic tissue of patients with chronic and autoimmune pancreatitis [37–40]. Furthermore, these data suggest the possibility that host dsDNA from tissue injury may be involved in the onset of the above-mentioned diseases via innate immunity activation. Aberrant MHC expression is not observed in pancreatic acinar cells but does exist in inter- and intralobular ductules [38]. PSCs are likely to be partially responsible for this expression. However, since it is likely that MHC class II may also be involved in the onset of autoimmune pancreatitis [40], stimulation other than host dsDNA may be related to the onset.

Since intracellular and extracellular dsDNA impaired the cell-specific function of PSC, functional loss of tissue repair was anticipated in environment where abundant necrotic dsDNA fragments were released from injured tissue and cells. Cell survival also decreased with intracellular dsDNA, which might recruit bone-marrow-derived PSC increasing the total number of PSC in the pancreas [41, 42]. It has been reported that engulfment of necrotic acinar cells attenuated the cell-specific function of PSC [6], which might reflect that the excessive amount of dsDNA in phagolysosome induced the leakage of dsDNA fragment and was recognized by cytosolic dsDNA sensor. We could not define the type of cell death because of the minimal effect of lipofectamine toward cell necrosis, but we thought that dsDNA induced both cell necrosis and apoptosis, which were often observed in pancreatitis.

**Figure 6:**
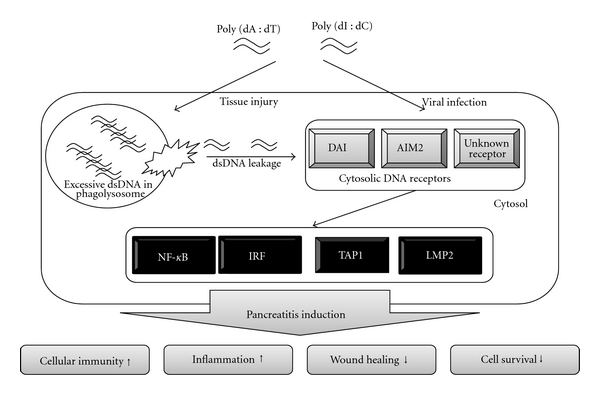
Model for mechanisms triggered by intracellular double-stranded DNA (dsDNA) in PSCs. The scheme depicts the induction of the innate immune response by dsDNA reflecting the onset and exacerbation of pancreatitis under sterile an nonsterile conditions.

In this study, we found that induction of the innate immune response by dsDNA reflects the onset and exacerbation of pancreatitis under sterile conditions (Figure 6). The results of this study will be very useful in elucidating the pathology of new pancreatitis and deciding on treatment targets for these diseases, including autoimmune pancreatitis. In the case of acute pancreatitis, DAMPs other than host dsDNA such as HSP [43] and uric acid [44] may be involved in the pathology; therefore, future studies should be performed from the viewpoint of the innate immunity.

## Figures and Tables

**Table 1:** Sequences of primers used in this study.

Gene	Sequence
Rat DAI: sense	5′- TGTCCCGCAGTAAAAGATGG -3′
Antisense	5′- TTCCAGCCAATGACAACCTC -3′
Rat AIM2: sense	5′- CATCACGGAGGAGGAACTGA -3′
Antisense	5′- CGTCCTGTCTGCAATGTTCA -3′
Rat TLR9: sense	5′- CCGAAGACCTAGCCAACCT -3′
Antisense	5′- TGATCACAGCGACGGCAATT -3′
Rat TNF-*α*: sense	5′- CTGGTGGTACCAGCAGATGG -3′
Antisense	5′- GGAGGCTGACTTTCTCCTGG -3′
Rat IL-6: sense	5′- CCACCAGGAACGAAAGTCAA -3′
Antisense	5′- CAGTCCCAAGAAGGCAACTG -3′
Rat MCP-1: sense	5′- ACGTGCTGTCTCAGCCAGAT -3′
Antisense	5′- GTTCTCCAGCCGACTCATTG -3′
Rat CINC-1: sense	5′- CCACACTCAAGAATGGTCGCG -3′
Antisense	5′- AGACGCCATCGGTGCAATC -3′
Rat NF-*κ*B	5′- TTCTGGGCCATATGTGGAGA -3′
p65: sense	
Antisense	5′- CCTCGCACTTGTAACGGAAA -3′
Rat RelB: sense	5′- GCCACGTAGCCTCTGAGTTG -3′
Antisense	5′- ATGGAGTGCTGGACCACAAG -3′
Rat IFN-*β*: sense	5′- TCCAGTTCCGACAAAGCACT -3′
Antisense	5′- CTTCCATTCAGCTGCCTCAG -3′
Rat IFN-*α*: sense	5′- TCTTCACACTCCTGGCACAAATG -3′
Antisense	5′- CTCTCAGTCTTCCCATCAAGTTGG -3′
Rat IRF1: sense	5′- GAGGGGACATCGAGATAGGC -3′
Antisense	5′- CTGGTAGAGTTGCCCAGCAG -3′
Rat IRF2: sense	5′- CCCGACATTGAGGAAGTGAA -3′
Antisense	5′- TTCTTGGAAGGTCGCTCAGA -3′
Rat IRF3: sense	5′- CCAGACCTGTCAACCTGGAA -3′
Antisense	5′- GGTCAAAAGGGTCCTTGCTC -3′
Rat IRF7: sense	5′- GCGACAAGGATCACCACATT -3′
Antisense	5′- CTCCAGCTTCACCAGGATCA -3′
Rat MHC I: sense	5′- GACACAGATCGCCAAGGGA -3′
Antisense	5′- ATATCCGCGGAGGAGGCT -3′
Rat MHC II: sense	5′5′- GAGGCGACCGTGTTTTCC -3′
Antisense	5′- TCTGTGACTGGCTTGCTGTT -3′
Rat TAP1: sense	5′- CCACCACATCCTCTTCCTCA -3′
Antisense	5′- ACCCTCCTCTCTCCATGAGC -3′
Rat LMP2: sense	5′- GGTGTAATGGGCAGAGGTGA -3′
Antisense	5′- AAGAATGGGAGGTGCTTGCT -3′
Rat *α*SMA: sense	5′- CCTCAGGGTGCTCGTGGAT -3′
Antisense	5′- CAGGACTGCCAGGCTCTCC -3′
Rat type I	5′- AGTTGGTGATGATGCCGTGTT -3′
collagen: sense	
Antisense	5′- ATGGGCCAAAAGGACAGCTAT -3′
Rat GAPDH: sense	5′- GCTCTCTGCTCCTCCCTGTT -3′
Antisense	5′- CACACCGACCTTCACCATCT -3′
